# Intravenous Amiodarone versus Digoxin in Atrial Fibrillation Rate Control; a Clinical Trial 

**Published:** 2017-01-10

**Authors:** Majid Shojaee, Bahareh Feizi, Reza Miri, Jalil Etemadi, Amir Hossein Feizi

**Affiliations:** 1 Emergency Department, Imam Hossein Hospital, Shahid Beheshti University of Medical Sciences, Tehran, Iran.; 2 Emergency Department, Bouali Hospital, Islamic Azad University, Tehran Medical Sciences Branch, Tehran, Iran.; 3 Cardiology Department, Imam Hossein Hospital, Shahid Beheshti University of Medical Sciences, Tehran, Iran.

**Keywords:** Amiodarone, digoxin, arrhythmias, cardiac, anti-arrhythmia agents, emergency department

## Abstract

**Introduction:**

Treatment of rapid ventricular response arterial fibrillation (rapid AF) varies depending on the decision of the in-charge physician, condition of the patient, availability of the drug, and the treatment protocol of the hospital. The present study was designed aiming to compare IV digoxin and amiodarone in controlling the heart rate of patients presenting to emergency department with rapid AF and relative contraindication for first line drug in this regard.

**Method:**

In the present clinical trial, patients presented to the ED with rapid AF and relative contraindication for calcium channel blockers and beta-blockers were treated with either IV amiodarone or IV digoxin and compared regarding success rate and complication using SPSS version 22. P < 0.05 was considered as statistically significant.

**Results:**

84 patients were randomly allocated to either amiodarone or digoxin treatment groups of 42 (53.6% male). The mean age of the studied patients was 61.8 ± 11.14 years (38 - 79). No significant difference was present regarding baseline characteristics. The rate of treatment failure was 21.4% (9 cases) in amiodarone and 59.5% (25 cases) in digoxin groups (p < 0.001). The mean onset of action was 56.66 ± 39.52 minutes (10 - 180) in amiodarone receivers and 135.38 ± 110.41 minutes (25 - 540) in digoxin group (p < 0.001). None of the patients showed any adverse outcomes of hypotension, bradycardia, and rhythm control.

**Conclusion:**

Based on the findings of the present study, rapid AF patients with relative contraindication for calcium channel blockers or beta-blockers who had received amiodarone experienced both higher (about 2 times) treatment success and a more rapid (about 2.5 times) response compared to those who received IV digoxin.

## Introduction

Arrhythmias are the causes of 12-20% of emergency department (ED) admissions and one of the most important differential diagnoses in patients with complaint of tachycardia, syncope, or chest pain (1). Atrial fibrillation (AF) is the most common supraventricular tachyarrhythmia found in 1-1.5% of the population (2, 3). Its prevalence rate is less than 0.5% in 40-50 year old population but reaches 5-15% in 80 year olds (4). In this dysrhythmia, re-entry is seen in both atriums and leads to ineffective atrial contraction (5). Clinical symptoms of AF include vertigo, tachycardia, chest pain, and fatigue and its risk factors include valvular and coronary heart diseases, cardiomyopathy, cardiac failure, alcohol and caffeine usage, thyrotoxicosis, anxiety, pheochromocytome, emotional stress, hypoxia, and etc. (5, 6). Calcium channel blockers and beta-blockers are the first line of treatment for this type of dysrhythmia in stable conditions. Digoxin is the second line due to limitations of using first line options in critically ill patients and elderly, and in presence of hypotension, kidney disorder, and heart failure (7). In addition, IV amiodarone has been used to safely restore the sinus rhythm and control heart rate in patients with stable hemodynamics and acute atrial tachy-dysrhythmia (7). Therefore, treatment varies depending on the decision of the in-charge physician, patient condition, availability of the drug, and the treatment protocol of the hospital. Based on the above-mentioned points, the present study was designed aiming to compare IV digoxin and amiodarone in controlling the heart rate of patients presenting to ED with rapid ventricular response AF (rapid AF) and relative contraindication for first line drugs in this regard.

## Methods

***Study design and setting:***

In the present clinical trial, patients presented to the ED of Imam Hossein, and Shohadaye Tajrish Hospitals, Tehran, Iran, with rapid AF and relative contraindication for calcium channel blockers and beta-blockers were treated with either IV amiodarone or IV digoxin and compared regarding success rate and complication. The study was approved by the Ethics Committee of Shahid Beheshti University of Medical Sciences and registered on Iranian Registry of Clinical Trials (IRCT) under the number IRCT201206047449N2. All patients filled informed consent forms before being included in the study. Researchers adhered to all recommendations of Helsinki protocol and confidentiality of patient information.

***Participants:***

Rapid AF patients aged between 18 and 80 years old, with stable vital signs, and any relative contraindication for first line treatments were included. Diagnosis of dysrhythmia was made based on 12-lead electrocardiogram by in-charge emergency physician and confirmed by a cardiologist. Patients with unstable hemodynamics, chest pain or shortness of breath, heart failure, and unconfirmed dysrhythmia, as well as those who had a history of allergy to the drug, underlying kidney or liver diseases, or had used anti-arrhythmic agents in the past 12 hours, were excluded. 

Documented or highly suspected evidence of heart failure and using either calcium channel blockers or beta-blockers were considered as the relative contraindications for first line treatment options. In addition, those who did not give their consent for participation in the study were not included. Since all patients would be under supervision for at least 12 hours after receiving the drug, those who did not want to stay in the hospital for that long were also excluded. 

***Intervention:***

Using block randomization, the included patients were randomly assigned to either IV amiodarone (Razi Company, Iran) or IV digoxin (Razi Company, Iran) group. To homogenize the groups regarding AF history, classified randomization was used. The amiodarone group patients were treated with 150 mg amiodarone diluted in 100 cc 5% dextrose water, intravenously infused during 10 minutes. In case of no improvement, another 150 mg dose was infused and all patients received a maintenance dose of 50 mg per hour during first 3 hours of treatment. For decreasing the probability of rhythm control, amiodarone was used with half dose, which is needed for rhythm conversion (150 mg instead of 300 mg). 

The other group was treated with 1 mg IV digoxin with initial injection of 0.5 mg and then two 0.25 mg doses in the second and fourth hour after intervention. 

The aim of treatment was control of heart rate and decreasing it to 80-100 beats per minute. Heart rate not being controlled with the mentioned doses was defined as treatment failure. All patients were under constant cardiac, respiratory, and vital signs monitoring. Due to differences in drug administration, follow-up and side effects between the two groups, the study could not be performed in a complete double-blind manner. Yet, drug administration, treatment evaluation, and data analysis were done by 3 separate people and the patients were blind to treatment. In this study, a heart rate over 110 per minute was defined as a rapid ventricular response. 

***Data gathering***

For all the studied patients a checklist consisting of demographic data (age, sex), history of digoxin consumption, time to treatment, presenting signs and symptoms, history of AF, and treatment outcomes (heart rate control and probable adverse outcome) was filled. Reduction of heart rate to less than 60 beats per minute, more than 20 mmHg drop in systolic blood pressure, and rhythm control were considered as adverse outcomes. Data gathering was done by a senior emergency medicine resident.

***Statistical analysis:***

Based on previous studies, treatment success was 31% in digoxin-treated group and 72% in amiodarone-treated group (8). Considering these rates and 95% confidence interval (95% CI) (α = 0.05), 90% power (β = 0.1) and 20% loss, sample size was calculated to be 41 patients. Considering intention to treat analysis, data were analyzed using SPSS version 22. Qualitative data were reported as frequency and percentage, and quantitative ones as mean ± standard deviation. To compare the studied variables between two groups, chi-square and t-test were used. P < 0.05 was considered as statistically significant.

## Results

***Baseline characteristics***

84 patients were randomly allocated to either amiodarone or digoxin treatment groups of 42 (53.6% male). The mean age of the studied patients was 61.8 ± 11.14 years (38 - 79). Table 1 shows the baseline characteristics of the patients. No significant difference was present regarding underlying diseases (p = 0.616), history of digoxin consumption (p = 0.641) and type of presentation on admission to ED (p = 0.189). 

***Outcome***

The rate of treatment failure was 21.4% (9 cases) in amiodarone and 59.5% (25 cases) in digoxin groups (p < 0.001). The mean onset of action was 56.66 ± 39.52 minutes (10 - 180) in amiodarone receivers and 135.38 ± 110.41 minutes (25 - 540) in digoxin group (p < 0.001). None of the patients showed any adverse outcomes of hypotension, bradycardia, and rhythm control.

**Table 1:** Baseline characteristics of the studied patients

**Variable **	**Amiodarone **	**Digoxin **	**P**
**Age (years)**	63.73 ± 11.06	59.88 ± 11.02	0.113
**Sex**			
Male	23 (54.76)	22 (52.38)	0.50
Female	19 (45.23)	20 (47.61)	
**Presenting sign and symptom**			
Dyspnea	20 (47.6)	14 (33.3)	0.189
Chest pain	13 (30.9)	9 (21.4)	
Palpitation	16 (38.1)	12 (28.5)	
Dizziness	1 (2.3)	2 (4.7)	
Others	2 (4.7)	10 (23.8)	
**History of digoxin consumption**			
Yes	30 (71.4)	27 (64.3)	0.641
No	12 (28.6)	15 (35.7)	

Data were presented as mean ± standard deviation, and frequency and percentage.

## Discussion

Based on the findings of the present study, rapid AF patients with relative contraindication for calcium channel blockers or beta-blockers who had received amiodarone experienced both higher (about 2 times) treatment success and a more rapid (about 2.5 times) response compared to those who received IV digoxin.

Currently, controlling heart rate is deemed more important than rhythm control in treating AF patients, especially in old people with non-acute symptoms (1). In 2008 Kirsten et al. discovered that the results of rate and rhythm control do not show significant difference regarding cardiovascular side effects, and prevalence of mortality, cardiac arrest, and cardiac failure (9). The effect of IV amiodarone in controlling the rate has been proved in most conditions such as cardiomyopathy and coronary artery and valvular diseases (2). Cochrane et al. compared the effects of amiodarone and digoxin in patients after cardiac surgery and found that administration of amiodarone as IV infusion after cardiac surgery is safe and as effective as digoxin, and decrease in rate is more obvious in amiodarone treated patients (2). In a study by Del Arco et al. in 2005, controlling the rate of the patients with amiodarone was more effective than digoxin (10). In our study also, response to treatment was higher in amiodarone treated group compared to digoxin group and treatment failure risk in amiodarone group was about half the digoxin group. 

Treatment failure in atrial tachy-dysrhythmia patients may have different reasons. Some believe that increase in sympathetic tone of the patients is the reason for not responding to some anti-arrhythmia drugs (7). In Heny et al. study, digoxin was ineffective in patients with increased sympathetic tone. In contrast, IV amiodarone was very effective and had high hemodynamic stability in critically ill patients (7). IV amiodarone has the ability to block calcium and sodium channels in addition to anti-androgenic characteristics. Its anti-androgenic characteristics increase due to its ability to decrease nor-epinephrine, and can therefore be more effective in cases of increased sympathetic tone. Based on the afore-mentioned points, it seems that between amiodarone and IV digoxin, amiodarone is a better choice for controlling the rate of AF patients who had relative contraindication for first line drugs of choice, in emergency settings. Yet, generalization of the findings has some limitations due to inability to double blind the study and small sample size.

## Conclusion:

Based on the findings of the present study, rapid AF patients with relative contraindication for calcium channel blockers or beta-blockers who had received amiodarone experienced both higher (about 2 times) treatment success and a more rapid (about 2.5 times) response compared to those who received IV digoxin.
